# Potential unfavorable impacts of BDNF Val66Met polymorphisms on metabolic risks in average population in a longevous area

**DOI:** 10.1186/s12877-016-0393-0

**Published:** 2017-01-05

**Authors:** Jun-Hua Peng, Cheng-Wu Liu, Shang-Ling Pan, Hua-Yu Wu, Qing-Hua Liang, Rui-Jing Gan, Ling Huang, Yi Ding, Zhang-Ya Bian, Hao Huang, Ze-Ping Lv, Xiao-Ling Zhou, Rui-Xing Yin

**Affiliations:** 1 Department of Pathophysiology, Guangxi Medical University, 22 Shuangyong Road, Nanning, 530021 Guangxi China; 2 Guangxi Colleges and Universities Key Laboratory of Human Development and Disease Research, 22 Shuangyong Road, Nanning, 530021 Guangxi China; 3 Department of Neurology, Jiangbin Hospital of Guangxi Zhuang Autonomous Region, 85 Hedi Road, Nanning, 530021 Guangxi China; 4 Department of Cardiology, The First Affiliated Hospital of Guangxi Medical University, 22 Shuangyong Road, Nanning, 530021 Guangxi China

**Keywords:** BDNF gene, Polymorphism, Metabolic risks, Longevity, Correlation

## Abstract

**Background:**

Brain-derived neurotrophic factor (BDNF) has been implicated in cognitive performance and the modulation of several metabolic parameters in some disease models, but its potential roles in successful aging remain unclear. We herein sought to define the putative correlation between BDNF Val66Met and several metabolic risk factors including BMI, blood pressure, fasting plasma glucose (FPG) and lipid levels in a long-lived population inhabiting Hongshui River Basin in Guangxi.

**Methods:**

BDNF Val66Met was typed by ARMS-PCR for 487 Zhuang long-lived individuals (age ≥ 90, long-lived group, *LG*), 593 of their offspring (age 60–77, offspring group, *OG*) and 582 ethnic-matched healthy controls (aged 60–75, control group, *CG*) from Hongshui River Basin. The correlations of genotypes with metabolic risks were then determined.

**Results:**

As a result, no statistical difference was observed on the distribution of allelic and genotypic frequencies of BDNF Val66Met among the three groups (all *P* > 0.05) except that AA genotype was dramatically higher in females than in males of CG. The HDL-C level of A allele (GA/AA genotype) carriers was profoundly lower than was non-A (GG genotype) carriers in the total population and the CG (*P =* 0.009 and 0.006, respectively), which maintained in females, hyperglycemic and normolipidemic subgroup of CG after stratification by gender, BMI, glucose and lipid status. Furthermore, allele A carriers, with a higher systolic blood pressure, exhibited 1.63 folds higher risk than non-A carriers to be overweight in CG (*OR* = 1.63, 95% *CI*: 1.05 - 2.55, P = 0.012). Multiple regression analysis displayed that the TC level of LG reversely associated with BDNF Val66Met genotype.

**Conclusions:**

These data suggested that BDNF 66Met may play unfavorable roles in blood pressure and lipid profiles in the general population in Hongshui River area which might in part underscore their poorer survivorship versus the successful aging individuals and their offspring.

**Electronic supplementary material:**

The online version of this article (doi:10.1186/s12877-016-0393-0) contains supplementary material, which is available to authorized users.

## Background

Brain-derived neurotrophic factor (BDNF) belongs to the neurotrophin family and plays essential roles in multiple processes associated with neuronal proliferation, differentiation and survival [1, 2]. It is mainly expressed in central nervous system (CNS) including the cortex, the hippocampus and limbic structures and is linked to better cognitive status [3]. In addition, BDNF can cross the blood-brain barrier in both directions, accounting for more than 70% of serum BDNF in healthy humans [4, 5]; serum BDNF concentration correlates closely with the cortical BDNF level and reflects the BDNF level in the brain [6].

Over the past decades, multiple lines of evidence have implicated that BDNF is not only stimulating for nerve growth and survival, but also exert nonneurotrophic effects over blood pressure, glucose, lipid and energy homeostasis in humans and model animals via central pathways involved in appetite regulation and energy expenditure [7–10]. It suppresses food intake, facilitates glucose uptake in the brain, reduces hepatic glucose production and converts white fat into brown fat in adipose tissue, leading to energy dissipation, lowered blood glucose and a lean phenotype; its downregulation in BDNF knockout mice and diabetic patients is associated with hyperphagic behavior, elevated blood glucose and cholesterol, and obese phenotype [10]. Together, these findings highlight the critical effects of BDNF on the maintenance of energy homeostasis and CNS functionality, perturbed BDNF signaling may confer metabolic risks which may further disrupt cognition and contribute to neurodegenerative pathologies such as Alzheimer’s disease (AD), depression and Parkinson’s disease (PD). It is thus reasonable to assume that long-lived individuals with better cognition might harbor beneficial BDNF genotypes and corresponding advantage profiles of metabolic parameters.

The human BDNF gene is located on the short arm of chromosome 11 at the boundary of 11p13 and 11p14 [11]. Several variants of BDNF gene have been reported and the most frequent is the functional Val66Met (c.196G > A, dbSNP: rs6265) polymorphism which substitutes a valine for a methionine at amino-acid residue 66 in the pro-BDNF protein, and the lower activity (Met) allele of this polymorphism is associated with reduced BDNF expression [12].

While BDNF Val66Met has been implicated as a genetic risk factor in several psychiatric disorders (e.g. schizophrenia, psychosis, major depression, anxiety, and eating disorders), cognitive compromised disorders (e.g. AD and PD) and metabolic syndrome [13–15], very little data are available on its association with metabolic risks in general population, especially in long-lived individuals with good cognitive function.

The longevous population dwelling along the Basin of Hongshui River in Guangxi Province of China is a representative cohort of successful aging who have maintained better cognition [16] and escaped most of the common aged-related disorders including hypertension, diabetes, AD and PD and have been established as a cohort for longevity research for decades [17]. Herein, we evaluated the putative association between BDNF Val66Met polymorphism and metabolic risk factors in this population sample of long-lived men and women. This work was presented at The 26th Great Wall International Congress of Cardiology/Asia Pacific Heart Congress 2015/International Congress of Cardiovascular Prevention and Rehabilitation 2015 [18].

## Methods

### Study subjects

The participants investigated in the present work joined the ongoing Bama Longevity Genetic Study (BLGS) which was initiated in 2008 as described previously [18]. Herein, the long-lived group (LG) included 487 (men 200, women 287) nonagenerians/centenarians (mean age ± SD, 93.28 ± 2.87 years) enrolled from native villages along Hongshui River Basin through Bama, Fengshan, Donglan and Du’an County of Guangxi Province, P. R. China. The offspring group (OG) comprised 593 natural children (son 333, daughter 260) of the studied nonagenerians/centenarians aged from 60 to 77 years (mean ± SD, 67.55 ± 8.02). The control group (CG) consisted 582 (men 328, women 254) unrelated elderly aged from 60 to 75 years (mean ± SD, 65.27 ± 9.32) recruited from the same residential area as the oldest olds without familial history of longevity. All subjects were of ethnically Zhuang, apparently healthy with normal cognition and had no evidence of any chronic disorder. They were on typical Zhuang diet whose staple foods included maize, rice, bean products, sweet potato and various local vegetables. The meat they ate and the oil they used for cooking were mainly pork and lard, respectively. All subjects could do as much farm work or home work (e.g. baby caring) as they could. Participants with a history of myocardial infarction, stroke, hypertension, diabetes, AD and PD and a history of medication taking (at least 4 weeks prior to our enrollment) were excluded. The current study was approved by the Ethics Committee of Guangxi Medical University. All participants could walk to the village clinics which were usually half a mile away from their houses where they were given verbal informed consents and their fingerprints (to express consent) were obtained after they received a full explanation of the study. Written informed consents were not obtained because of their low educational level. The consent procedure was also approved by the Ethics Committee of Guangxi Medical University. An incentive of about ten dollars was provided to each participant in the study [19].

### Epidemiological survey

Information on socio-demography and lifestyles was collected with standardized questionnaires. Anthropometric parameters including blood pressure, body height, body weight, waist circumference and body mass index (BMI) were measured or calculated in all groups as described previously [20, 21]. Hypertension was defined as systolic blood pressure (SBP) > 140 mmHg and/or diastolic blood pressure (DBP) > 90 mmHg, and/or previous usage of anti-hypertensive medications prior to the survey. Normal weight, overweight, and obesity were defined as BMI < 24, 24 - 28, and > 28 kg/m^2^, respectively [20, 21].

### Biochemical analyses

An overnight fasting venous blood sample of 8 mL was collected for each subject, 4 mL of which was for serum separation and subsequent serological detections while the remaining was for DNA extraction. Fasting plasma glucose (FPG) was measured immediately by a blood glucose meter (Accu-Chek Active, Roche, Germany). Total cholesterol (TC), triglycerides (TG), low density lipoprotein cholesterol (LDL-C) and high density lipoprotein cholesterol (HDL-C) levels were assayed by enzymatic methods as previously described [20]. Individuals with FPG > 7.1 mmol/L and TC > 5.17 mmol/L and/or TG > 1.70 mmol/L were defined as hyperglycemia and dyslipidemia, respectively [21].

### Sample preparation and genotyping

Isolation of genomic DNA of leukocytes was performed according to standard procedures. Genotyping of BDNF Val66Met polymorphism was conducted by amplification refractory mutation system-polymerase chain reaction (ARMS-PCR) as described by Sheikh et al. [22]. The key principle for this methodology is that oligonucleotides with a mismatched 3’ residue do not function as a primer in the PCR due to the lack of 3’ exonucleolytic activity of Taq DNA polymerase. In brief, two sets of PCR primers were used in the PCR where the first set (P1, forward, 5′-CCTACAGTTCCACCAGGTGAGAAGAGTG-3′ and P2, reverse, 5′-TCATGGACATGTTTGCAGCATCTAGGTA-3′) was expected to the 401 bp region containing the SNP of interest, while the second set (P3 and P4, 5′-CTGGTCCTCATCCAACAGCTCTTCTATaAC-3′ and 5′-ATCATTGGCTGACACTTTCGAACcCA-3′, in which lower case letter indicated mismatched nucleotide) was allele specific and accounted for the G → A substitution. PCR amplifications were carried out in a 20 μL reaction volume containing 25 ng of template DNA, primers (P1, P2, P3 and P4, 1.25 μmol/L for each) and 10 μL of 2× Taq Master Mix (Beijing ComWin Biotech Co.,Ltd, China) and were run in a Biometra PCR system (TProfessional standard, Germany) with a predenaturation at 94 °C for 5 min, followed by 30 cycles of 94 °C for 45 s, 62.5 °C for 60 s and 72 °C for 60 s and a final extension at 72 °C for 5 min. PCR products (8 μL) were resolved on a 2% agarose gel for 30 min at 80 V and stained with Golden View I (Beijing Solorbio Co., Ltd, China). The 401 bp band represented the control amplicon, whereas the 253 and 201 bp bands represented the G and A allele-specific amplicons. For the genotyping reliability, thirty randomly selected DNA samples (ten for each genotype) were sequenced and the sequencing results were all in agreement with that of PCR genotyping. Laboratory technicians who performed genotyping were masked to clinical and serological data.

### Statistical analyses

All data were analyzed using the SPSS 16.0 statistical software package (SPSS Inc, Chicago, IL). Levels of quantitative variables were presented as mean ± SD except that TG levels were in medians (interquartile) due to skewed distribution. Allelic and genotypic frequencies were calculated directly. Hardy-Weinberg equilibrium was computed for the expected genotype distribution using the standard goodness-of-fit test. Difference in genotypic and allelic distribution between groups was estimated with chi-square test, and odds ratio (OR) and 95% confidential interval (CI) were used to indicate relative risk. The statistical evaluation for the categorical variables between groups was assessed by one-way ANOVA test. The association of BDNF genotypes with blood pressure, BMI, FPG and serum lipid variables was evaluated using analysis of covariance (ANCOVA). Multiple logistic analyses with stepwise modelling were used to evaluate the association of blood pressure, BMI, FPG and serum lipid levels with genotypes (GG = 1, GA = 2, AA = 3) and several environment factors. In all hypothetical tests, two-tailed values of *P* < 0.05 were considered statistically significant.

## Results

### Population characteristics and metabolic measures

Table 1 summarizes the demographic, clinical, and biochemical characteristics of the three groups investigated. OG and CG did not differ in terms of age (*P* > 0.05). After adjustment for age and sex, DBP, TC, and LDL-C were found to be higher while FPG was lower in LG as compared with OG and/or CG. The blood levels of FPG, HDL-C and LDL-C of OG were significant higher than that of CG.

**Table 1:** Population characteristic and metabolic parameters of participants

Parameter	LG (*n* = 487)	OG (*n* = 593)	CG (*n* = 582)	*F(χ* ^*2*^ *)*	*P*
Age (years)	93.28 ± 2.87*^▲^	58.35 ± 9.06	58.18 ± 9.77	3239.680	0.000
Male/Female	200/287	333/260	328/254	31.813	0.000
WC (cm)	76.23 ± 9.14	75.08 ± 9.27	75.53 ± 9.41	0.340	0.712
BMI (kg/m^2^)	20.21 ± 3.63	21.68 ± 3.00	21.69 ± 3.24	0.022	0.978
FPG (mmol/L)	4.65 ± 1.18^▲^	4.97 ± 1.80*	4.73 ± 1.52	8.070	0.000
SBP (mm Hg)	165.83 ± 26.15	137.81 ± 22.96	136.02 ± 23.56	0.618	0.539
DBP (mm Hg)	89.21 ± 13.17*^▲^	85.15 ± 11.22	84.68 ± 11.31	4.020	0.018
PP (mm Hg)	76.61 ± 23.74	52.66 ± 19.81	51.34 ± 18.40	2.484	0.084
TC (mmol/L)	5.09 ± 1.00*	5.31 ± 1.13*	4.88 ± 1.00	28.690	0.000
TG (mmol/L)	0.97(0.45)	1.00(0.89)	0.95(0.66)	2.150	0.117
HDL-C (mmol/L)	1.61 ± 0.53	1.71 ± 0.57*	1.62 ± 0.50	3.353	0.035
LDL-C (mmol/L)	2.99 ± 0.87*	2.99 ± 1.04*	2.61 ± 0.92	27.325	0.000

*Abbreviations*: *LG* long-lived group, *OG* offspring group, *CG* control group, *WC* waist circumference, *BMI* body mass index, *FPG* fasting plasma glucose, *SBP* systolic blood pressure, *DBP* diastolic blood pressure, *PP* pulse pressure, *TC* total cholesterol, *TG* triglyceride (presented as median and interquartile range), *HDL-C* high-density lipoprotein cholesterol, *LDL-C* low-density lipoprotein cholesterol

^▲^ *vs* OG, *P* <0.05; *vs CG, *P* <0.05

### Genetic distribution

**Fig. 1:**
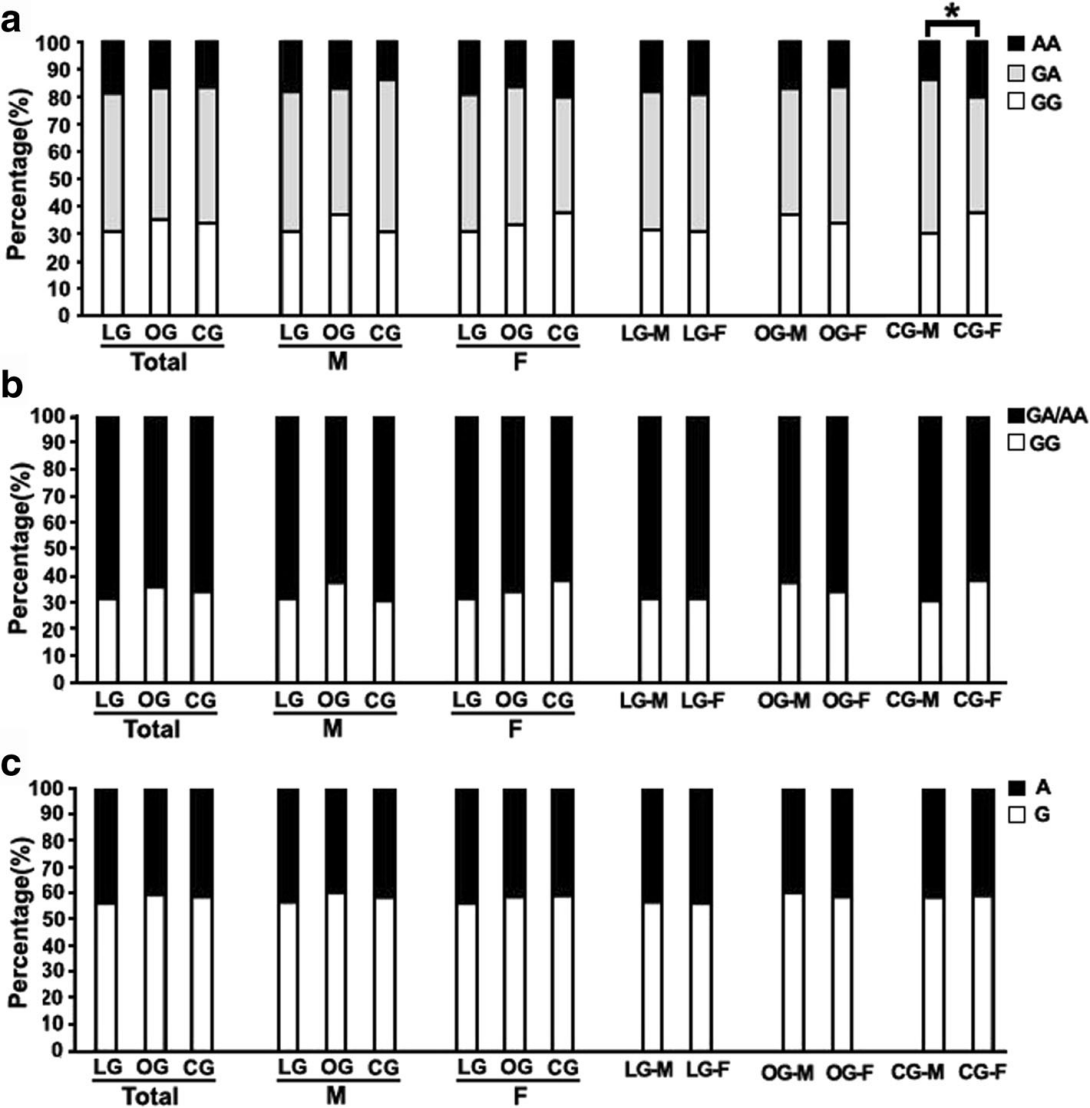
Comparison of genotypic and allelic frequencies of the BDNF Val66Met among groups. **a** Genotypic frequency under co-dominant model, females in CG represented more AA genotype than males (Chi-square test, *χ* ^*2*^ = 11.263,*P* = 0.004). **b** Genotypic frequency under dominant model, no difference among groups and between sexes. **c** Allelic frequency, no difference among groups and between sexes. *P < 0.05. Abbreviations: LG, long-lived group; OG, offspring group; CG, control group; M, males; F, females

The distribution of the BDNF Val66Met alleles fit the Hardy-Weinberg equilibrium. Its minor allele frequency (MAF) in LG, OG and CG was 43.3%, 41.1% and 41.9 respectively. Overall, the distribution of genotypic and allelic frequency of this SNP did not differ across groups (*P* > 0.05) except that females represented more AA genotype than males (*P* = 0.004, Fig. 1a) in control group under co-dominant but not dominant model, which persisted through sex stratification (*P* > 0.05 for all, Fig. 1a, b and c).

### Genotypes and metabolic risk factors

**Fig. 2:**
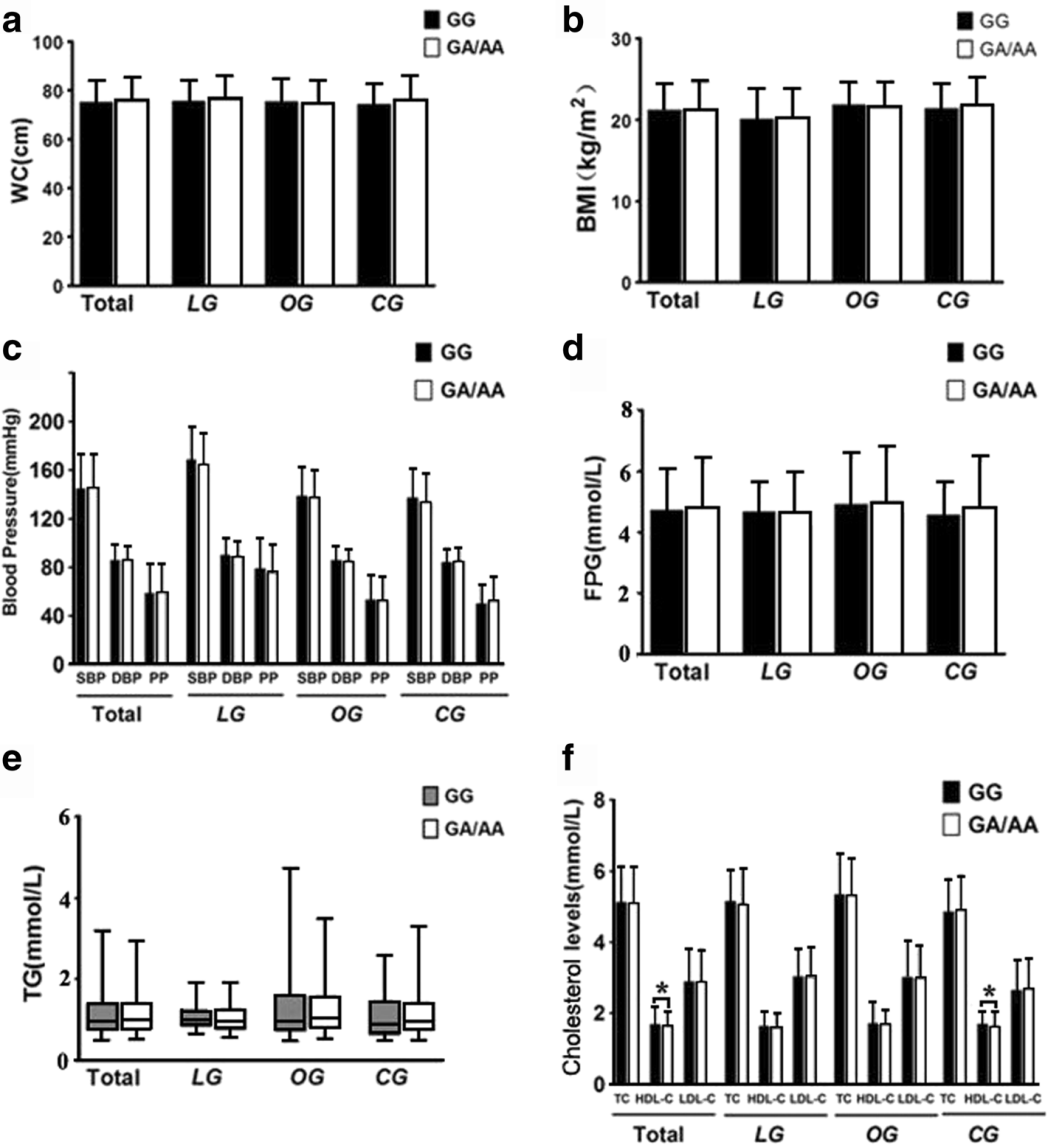
Overall association of BDNF Val66Met genotypes with metabolic risk parameters. (**a**) with WC; (**b**) with BMI; (**c**) with blood pressure; (**d**) with FPG; (**e**) with TG; (**f**) with cholesterol levels. TG value was presented as Whiskers 5-95 percentile while the values of other parameters were presented as Mean ± SD. * indicates *P* < 0.05. Abbreviations: LG, long-lived group; OG, offspring group; CG, control group; WC, waist circumference; BMI, body mass index; FPG, fasting plasma glucose; SBP, systolic blood pressure; DBP, diastolic blood pressure; PP, pulse pressure; TC, total cholesterol; TG, triglyceride; HDL-C, high-density lipoprotein cholesterol; LDL-C, low-density lipoprotein cholesterol

**Fig. 3:**
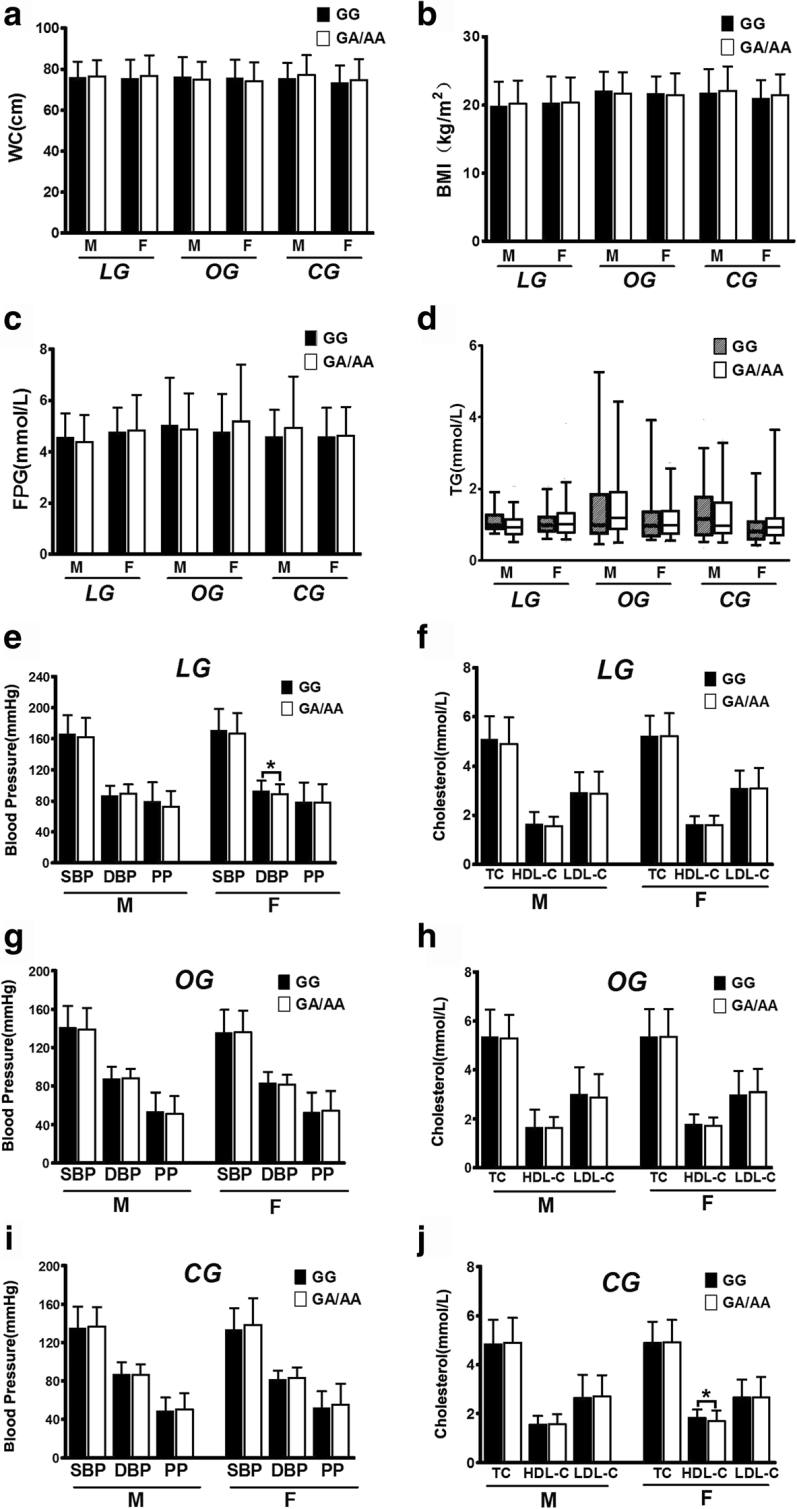
Association of BDNF Val66Met genotypes with metabolic risk parameters stratified by sex. With WC (**a**), BMI (**b**), FPG (**c**) and TG (**d**) in the three groups investigated; with blood pressure and cholesterol levels in LG (**e** and **f**, respectively), in OG (**g** and **h**, respectively) and in OC (**i** and **j**, respectively). Not all association panels is presented in this figure. M, males; F, females. Other notes and abbreviations see legends of Fig. 2

Overall, no difference was observed between the impact of BDNF rs6265 A genotype (GA/AA) and non-A genotype (GG) on waist circumference (WC), BMI, FPG, TG and blood pressure in combined population and the three groups under investigation (Fig. 2a, b, c, d, and e) except that HDL-C level of A genotype carriers was dramatically lower than non-A carriers in the combined population (*P* = 0.009) and in the controls (*P* = 0.006) (Fig. 2f). These trends did not change basically through sex stratification (Fig. 3a-j). When BMI, FPG and lipid status were taken into consideration, the SBP level of A genotype carriers was noticeably higher (*P* = 0.023) (Additional file 1: Figure S1, I) while HDL-C was lower (*P* = 0.003) (Additional file 1: Figure S1, J) than non-A carriers in the normal BMI subgroups of the controls; lowered FPG level in the euglycaemic subfraction of LG (*P* = 0.038) (Additional file 2: Figure S2, C), increased SBP level in the hyperglycemic subfraction of OG (*P* = 0.041) (Additional file 2: Figure S2, G) and lowered HDL-C level in the hyperglycemic subfraction of CG (*P* = 0.001) (Additional file 2: Figure S2, J) were found in individuals harboring A genotype versus non-A genotype; a reduced FPG concentration in LG normolipidemic subclass (*P* = 0.025) (Additional file 3: Figure S3, C), a reduced BMI and HDL-C (*P* = 0.014 and *P* = 0.004, respectively) (Additional file 3: Figure S3, B and H) in OG normorlipidemic subclass and a raised BMI level (*P* = 0.008) (Additional file 3: Figure S3, B) while a compromised HDL-C level (*P* = 0.031) (Additional file 3: Figure S3, J) in CG normorlipidemic subclass were detected when A genotype bearers were compared with non-A genotype bearers. Relative risk estimates showed that allele A carriers have 1.63 folds higher risk to be overweight in CG (*OR* = 1.63, 95% *CI* : 1.05 - 2.55) while exhibit less risk to be hyperglycemic (*OR* = 1.039, 95% *CI* = 0.619 - 1.742) and hyperlipidemic (*OR* = 1.198,95% *CI* = 0.847 - 1.694) in LG, OG and CG as compared with non-A carriers. Together, these data mainly demonstrate that the mutant Met allele of BDNF gene may have different impact on metabolic parameters in different population and these impacts might be influenced by different BMI, FPG and lipid status.

### Correlation analyses

Multiple linear regression analyses revealed that BDNF Val66Met genotype was reversely correlated with TC level in LG. No correlation was observed between BDNF Val66Met genotype and other metabolic parameters in the three studied groups (Table 2).

**Table 2:** Correlation between parameters and the BDNF Val66Met polymorphism

Parameter	Relative factor	Unstandardized coefficients	Standard error	Standardized coefficients	*t*	*P*
LG						
BMI	WC	0.118	0.020	0.296	5.924	0.000
	HC	0.184	0.025	0.342	7.333	0.000
	DBP	−0.498	0.227	−0.072	−2.193	0.029
FBG	Sex	0.353	0.106	0.147	3.326	0.001
	TG	0.391	0.122	0.143	3.217	0.001
	WC	0.012	0.006	0.093	2.094	0.037
SBP	Sex	4.815	2.401	0.091	2.005	0.045
DBP	TC	1.879	0.586	0.143	3.208	0.001
	BMI	0.484	0.162	0.133	2.995	0.003
PP	-	-	-	-	-	-
TC	DBP	0.011	0.003	0.139	3.146	0.002
	Sex	0.223	0.091	0.110	2.445	0.015
	FBG	0.088	0.038	0.103	2.306	0.022
	Genotype	−0.132	0.063	−0.093	−2.090	0.037
TG	FBG	0.058	0.016	0.159	3.556	0.000
	DBP	0.004	0.001	0.108	2.425	0.016
HDL-C	BMI	−0.015	0.007	−0.105	−2.336	0.020
LDL-C	DBP	0.009	0.003	0.131	2.930	0.004
	Sex	0.230	0.079	0.131	2.921	0.004
OG						
BMI	WC	0.090	0.037	0.054	2.427	0.016
	Sex	0.730	0.144	0.121	5.062	0.000
	DBP	0.040	0.006	0.148	6.502	0.000
	Age	0.171	0.067	0.059	2.554	0.011
FBG	WC	−0.041	0.011	−0.173	−3.691	0.000
	DBP	−0.649	0.131	−0.205	−4.952	0.000
SBP	Age	0.902	0.096	0.356	9.377	0.000
	BMI	1.522	0.290	0.199	5.251	0.000
	Sex	−4.215	1.751	−0.091	−2.407	0.016
DBP	BMI	1.669	0.172	0.447	9.717	0.000
	Sex	−4.944	0.838	−0.219	−5.897	0.000
	HC	−0.924	0.421	−0.085	−2.197	0.028
	FBG	−0.651	0.235	−0.105	−2.767	0.006
PP	Age	0.859	0.083	0.393	10.389	0.000
TC	WC	−0.037	0.013	−0.213	−2.911	0.004
	Age	−0.013	0.005	−0.105	−2.670	0.008
	BMI	0.069	0.027	0.182	2.588	0.010
	DBP	−0.008	0.004	−0.084	−2.065	0.039
TG	WC	0.057	0.011	0.353	5.282	0.000
	Sex	−0.076	0.033	−0.151	−2.287	0.023
HDL-C	WC	−0.012	0.002	−0.189	−4.676	0.000
	Sex	0.115	0.045	0.100	2.543	0.011
LDL-C	BMI	0.113	0.014	0.328	7.871	0.000
	DBP	−0.012	0.004	−0.125	−2.996	0.003
CG						
BMI	WC	0.151	0.025	0.278	5.998	0.000
	Age	0.247	0.079	0.084	3.123	0.002
FBG	WC	0.186	0.055	0.137	3.359	0.001
SBP	Age	−4.021	1.801	−0.086	−2.232	0.026
	HC	0.706	0.152	0.181	4.631	0.000
DBP	Sex	0.541	0.142	0.157	3.822	0.000
	FBG	−0.650	0.305	−0.087	−2.133	0.033
PP	Age	−3.783	1.391	−0.104	−2.720	0.007
	HC	0.429	0.114	0.141	3.751	0.000
	Sex	4.185	1.404	0.113	2.980	0.003
TC	BMI	0.045	0.020	0.149	2.310	0.021
	Age	−0.015	0.004	−0.143	−3.320	0.001
	CC	−0.040	0.011	−0.275	−3.737	0.000
	WC	0.026	0.008	0.243	3.117	0.002
TG	BMI	0.076	0.021	0.223	3.586	0.000
	Age	−0.027	0.005	−0.233	−5.740	0.000
	FBG	0.096	0.029	0.131	3.270	0.001
	HC	−0.058	0.013	−0.315	−4.616	0.000
	WC	0.029	0.009	0.246	3.365	0.001
HDL-C	Sex	−0.003	0.001	−0.108	−2.688	0.007
	WC	−0.008	0.002	−0.154	−3.827	0.000
LDL-C	WC	0.012	0.004	0.120	2.914	0.004

*Abbreviations: LG* long-lived group, *OG* offspring group, *CG* control group, *WC* waist circumference, *HC* hip circumference, *CC* chest circumference, *BMI* body mass index, *FPG* fasting plasma glucose, *SBP* systolic blood pressure, *DBP* diastolic blood pressure, *PP* pulse pressure, *TC* total cholesterol, *TG* triglyceride, *HDL-C* high-density lipoprotein cholesterol, *LDL-C* low-density lipoprotein cholesterol

## Discussion

Instead of finding salutary genotypes of BDNF gene which may account in part for better preservation of health in the oldest olds and their offspring, we noted unexpectedly an overrepresentation of BDNF 66 Met and its adverse correlation with several metabolic parameters in the general population living in the same area as the long-lived families, which can also explain why local residents have higher morbidity and mortality as compared to individuals with exceptional longevity. These results are in concert with some but not all observations from other research groups. Here, the effect of this SNP on several common metabolic risks is discussed basing on our data.

### BDNF Val66Met and obesity

Obesity has been so common and has emerged as a worldwide health problem due to its association with increased risk of metabolic-related disorders. BMI is one of the most important indicators for obesity assessment. Several previous studies had implicated that the derived Met allele of BDNF gene might down-regulate BDNF, raise blood glucose concentration and predispose individuals to obesity [12, 13, 23, 24]. In the present work, BDNF Met allele carriers with normal BMI and lipid levels in OG displayed lower BMI whereas Met carriers with normal lipids in CG exhibited higher BMI level and risk of overweight, demonstrating a different impact pattern of BDNF Met on body shape between the offspring of nonagenarians and the age-matched controls. These findings are in accordance with those in several previous studies but not in others. For instance, Estonian adolescent girls bearing BDNF Val allele tended to have lower BMI than who bear Met allele; Croatian Caucasian children and adolescents who presented one or two Met alleles were prone to obesity; Puerto Rican women living in Boston with the GA or AA genotype were 50% more likely to be overweight compared to GG carriers [25–27]. Mounting experimental evidence supports this association of Met variant with obesity: the derived Met allele reduces the expression of BDNF, which plays pivotal roles in the inhibition of excess calorie uptake (e.g. vigorous appetite, bad dietary behavior and sedentary lifestyle) and in the enhancement of energy expenditure (e.g. acceleration of brown fat metabolism and the degeneration of serum level of TG, TC, and free fatty acids and improvement of insulin resistance) [23, 28, 29]. On the contrary, Met allele was either linked to thin phenotype in British females, Chinese Han adolescents and Korean smokers [30–32] or lacked of relationship with obesity in German extremely obese children and adolescents and underweight students [33]. The inconsistency of these results indicates the uncertain association between BDNF Val66Met and obese phenotype which might be modulated by other factors such as ethnic background, age, gender, living environment, dietary structure and lifestyle [34]. Intriguingly, the Met allele carriers in OG exhibited a lower BMI level, implying that the descendants of nonagenarians may endow other unraveled buffering genotypes which may attenuate the unfavorable effect of this variant. In addition, the individuals with Met allele in the overweight subclass of CG presented a higher waist circumference, indicating that Met is mainly linked to abdominal obesity.

### BDNF and blood pressure

Emerging evidences show that BDNF might be involved in the regulation of arterial pressure via different pathways. For instance, it has been recently demonstrated that long-term elevation of BDNF in the paraventricular nucleus of the hypothalamus has a major impact on central regulation of sympathetic activity, blood pressure and heart rate which is mediated by ANG II/angiotensin type 1 receptor and angiotensin/Mas [35]. Dietary high salt intake promotes vasopressin release which contributes to the elevation of arterial pressure through BDNF-TrkB-GABAergic signaling system [35–37]. BDNF Val66Met modulates hypothalamic-pituitary-adrenal axis reactivity and regulation, with women bearing Val/Met genotype and men bearing Val/Val being particularly vulnerable to psychological stress [38], an essential etiology for primary hypertension. These data implicate that higher central or circulating BDNF may account for arterial hypertension and Met variant may link to a reduced BDNF level and is favorable to better blood pressure regulation. However, we detected an elevated but not lowered SBP level in A genotype carriers in the normal BMI subgroups of the controls and an elevated DBP level in the hyperglycemic subgroup of OG when compared to non-A genotype carriers. We have no further explanation for this result due to the lack of serum BDNF level and other functional experiments on this polymorphism.

### BDNF Val66Met and glucose metabolism

As mentioned above, previous studies on model animals had demonstrated that BDNF may suppress food uptake, improve insulin resistance and maintain glucose homeostasis [28, 29]. Intracerebroventricular or intraperitoneal administration of BDNF results in a reduction of plasma glucose level of diabetic or insulin deficient mice [10]. Clinical epidemiological investigation also observed that the serum BDNF level of diabetic patients were significantly lower than that of normal controls and were reversely correlated with blood glucose concentration independent of obesity, indicating the robust relationship of BDNF and glucose metabolism in that the hyperglycemic status of diabetic patients might have repressed the release of BDNF from the brain [39, 40]. Furthermore, male BDNF Met/Met carriers with clozapine-induced metabolic syndrome exhibited significant higher fasting glucose levels than those with Val/Val or Val/Met genotypes [14]; carriers of Met allele displayed elevated blood glucose and predicted lower memory scores [41]. These observations demonstrated a pronounced influence of BDNF on glucose homeostasis. However, Bonaccorso et al. [13] detected no different glucose level between Met and non-Met carriers in patients with bipolar and schizoaffective disorder, indicating the uncertainty affecting pattern of BDNF on glucose metabolism. In the present study, a lowered FPG was only noted in the BDFN GA/AA carriers of euglycemic and normolipidemic subgroup of LG but not in any genotype of other groups, implying a limited effect of BDNF rs6265 on blood glucose modulation in our studying populations. The relatively lower average FPG levels and lower hyperglycemia prevalence in these populations might be one of the explanations for this observation.

### BDNF Val66Met and lipid metabolism

Information regarding the influence of BDNF on lipid modulation is scarce. Tsuchida and colleagues were some of the researchers who firstly focused on the effect of BDNF on lipid metabolism and noted that subcutaneous injection of BDNF (twice/week) could significantly improve lipid and glucose profiles in diabetic mice [42]. In humans, serum BDNF levels were found to be significantly higher in type 2 diabetic patients than in the healthy controls and positively correlated with triglyceride level [43]. As compared to non-Met, BDNF 66Met carriers with bipolar disorder showed a greater increase in triglycerides and triglycerides/HDL-C ratio after three or six months of antipsychotic therapy [13]. We noted herein similar correlation trend between BDNF Val66Met and lipid profiles where Met allele was essentially linked to elevated triglyceride but lowered HDL-C level relative to non-Met bearers, particularly in the control and offspring groups. Tsuchida et al. postulated that BDNF might enter the brain and act on the hypothalamic neurons, leading to activation of sympathetic nerve system, enhancement of energy expenditure and reduction of lipid accumulation as does leptin [42]. It is thus reasonable to assume that the derived Met allele may account for a lower BDNF level and a reduced energy consumption and lipid increment in our participants.

### BDNF Val66Met and neuroinflammation

Normal aging has been regarded as a chronic and low-grade pro-inflammatory state, with an over-expression of systemic inflammatory cytokines including IL-1, IL-6 and TNF-α etc. A number of highly prevalent risk factors such as obesity, diabetes, hypertension, and atherosclerosis are increasingly believed to silently contribute to systemic- and neuro-inflammation [44–46]. Cerebral small vessel disease and perfusion deficits resulting from these pathological conditions may account for the neuroinflammation and subsequent neurodegeneration and cognitive dysfunction [46]. BDNF has been implicated to play a neuroprotective role in the central nerve system; low levels of BDNF have been associated with aging and an array of neurological and psychiatric disorders [47, 48]. One of the explanations for this protection in that it counteracts the effects of some inflammatory cytokines such as C-reactive protein [49]. It is thus reasonable to assume that any BDNF polymorphism with lower BDNF activity may link to enhanced neuroinflammation and worsened cognitive outcome. This notion is underscored by the observation of Dooley and colleagues who noted a positive association between C-reactive protein levels and cognitive depressive symptoms among BDNF Met carriers of breast cancer survivors [50]. Therefore, we postulate that the control females who were enriched with BDNF Met allele in our study may also have higher inflammatory cytokines and poorer cognition, however this needs to be confirmed in our future longitudinal follow-up.

### Limitations

The first limitation of the present study is that we only focused on BDNF genotype, without assessing the circulating BDNF protein levels. There are several intervening aspects between an individual’s genotype and its function, therefore an individual’s BDNF genotype does not necessarily determine BDNF protein levels. Although previous study has shown a correlation between the Val66Met polymorphism and circulating BDNF level [51], indicating that BDNF genotype may serve as a proxy for protein levels at least in some samples, others have not found this association [52]. The second limitation is that we failed to detect inflammatory biomarkers (e.g. CRP, IL-1, 6, TNF-α) which are very important parameters in evaluating the association of BDNF genotype with neuroinflammaging. Finally, we failed to collect data on cognitive status (e.g. MMSE score) and life style (e.g. dietary habit, alcohol consumption, smoking and physical activity) which may be potential confounders on our results. All these parameters and data will be considered in our future research on these populations.

## Conclusions

In conclusion, the key finding of the current work is that BDNF Met variant represents more frequently and correlates with elevated BMI and SBP levels and lowered HDL-C level in the average population inhabiting the river basin of Hongshuihe, which may be some of the contributors to their poorer survivorship as compared to whom achieve prolonged longevity. Further investigations on the interactions among serum BDNF and insulin levels and metabolic measures are warranted.

## Additional files

Additional file 1: Figure S1. Association between BDNF Val66Met genotypes and metabolic risk parameters stratified by BMI status, N, normal; OW, overweight; other notes and abbreviations see legends of Fig. 3. (JPG 1311 kb)
Additional file 2: Figure S2. Association between BDNF Val66Met genotypes and metabolic risk parameters stratified by FPG status, E, euglycaemia; H, hyperglyceamia. Other notes and abbreviations see legends of Fig. 3. (JPG 1271 kb)
Additional file 3: Figure S3. Association between BDNF Val66Met genotypes and metabolic risk parameters stratified by lipid status, N, normolipidemia; H, hyperlipidemia. Other notes and abbreviations see legends of Fig. 3. (JPG 1289 kb)

## Abbreviations

AD: Alzheimer’s disease
BDNF: Brain-derived neurotrophic factor
BMI: Body mass index
CI: Confidential interval
CNS: Central nervous system
DBP: Diastolic blood pressure
FPG: Fasting plasma glucose
HDL-C: High density lipoprotein cholesterol
LDL-C: Low density lipoprotein cholesterol
OR: Odds ratio
PD: Parkinson’s disease
SBP: Systolic blood pressure
TC: Total cholesterol
TG: Triglycerides
