# Supplementary figures and images for: Potential unfavorable impacts of BDNF Val66Met polymorphisms on metabolic risks in average population in a longevous area
Source: BMC Geriatr. 2017 Jan 5;17:4. doi: 10.1186/s12877-016-0393-0 (PMC5217242; doi:10.1186/s12877-016-0393-0)

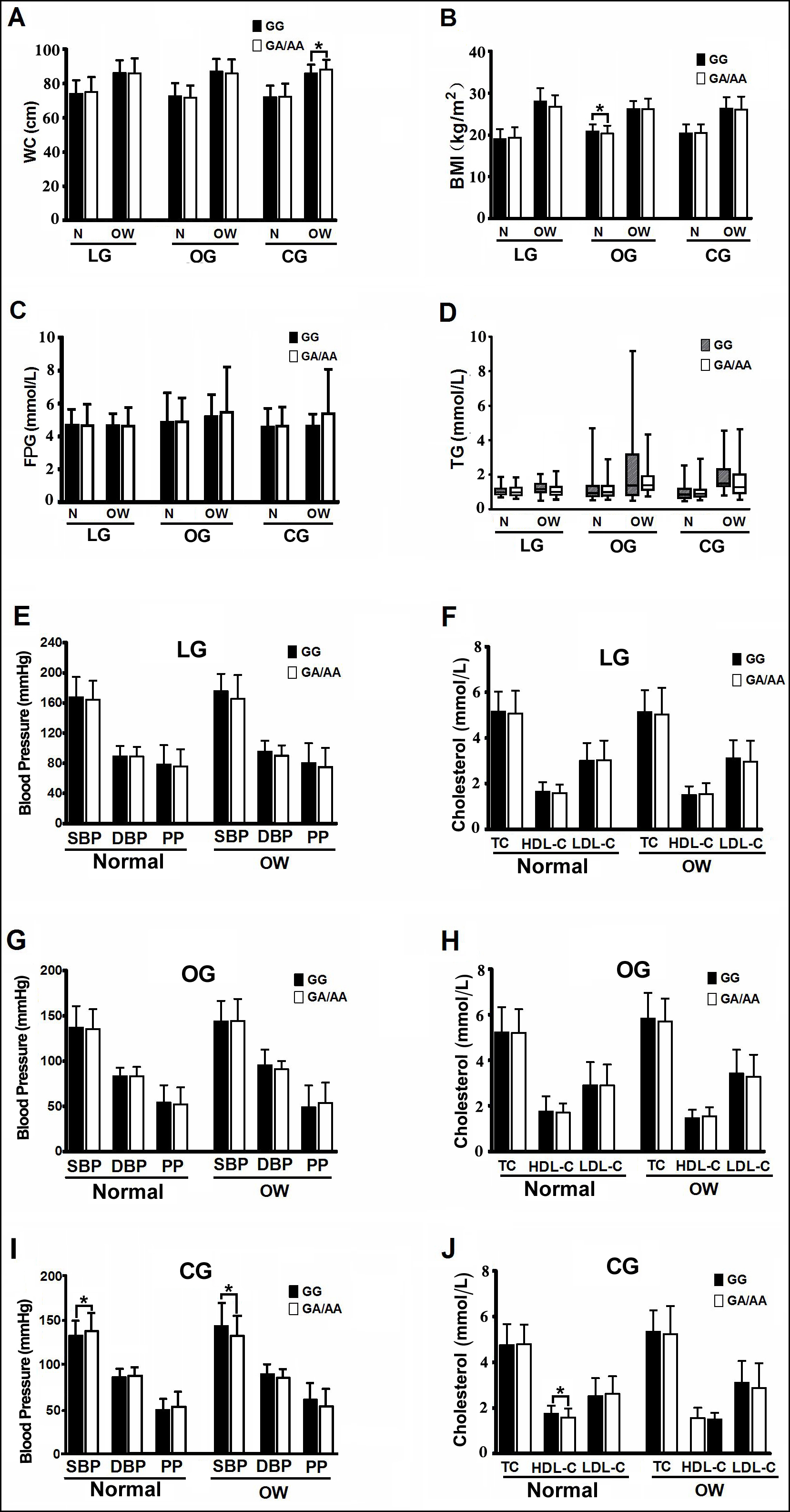

Supplement: Additional file 1: Figure S1. — Association between BDNF Val66Met genotypes and metabolic risk parameters stratified by BMI status, N, normal; OW, overweight; other notes and abbreviations see legends of Fig. 3. (JPG 1311 kb) [file 12877_2016_393_MOESM1_ESM.jpg]

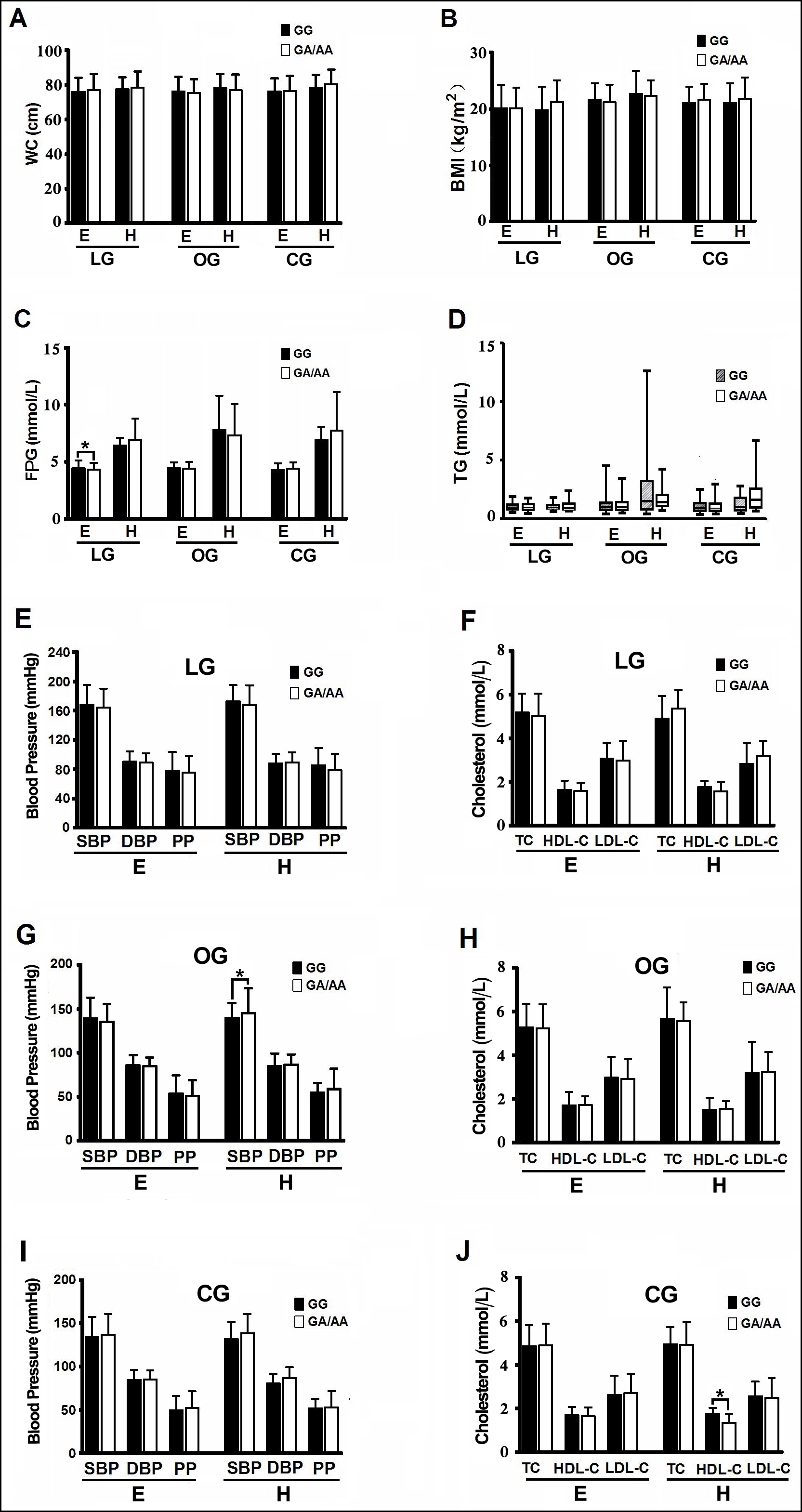

Supplement: Additional file 2: Figure S2. — Association between BDNF Val66Met genotypes and metabolic risk parameters stratified by FPG status, E, euglycaemia; H, hyperglyceamia. Other notes and abbreviations see legends of Fig. 3. (JPG 1271 kb) [file 12877_2016_393_MOESM2_ESM.jpg]

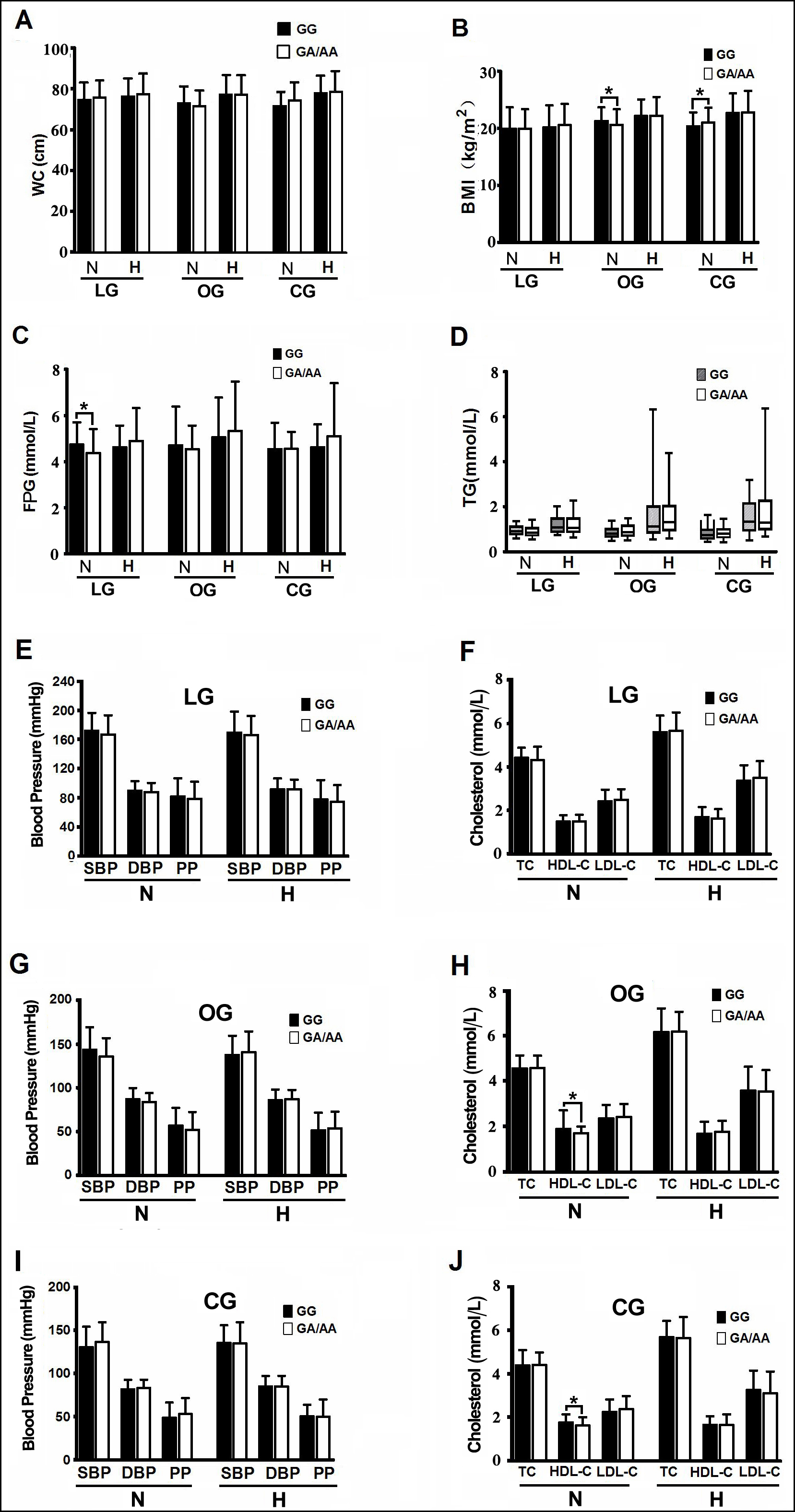

Supplement: Additional file 3: Figure S3. — Association between BDNF Val66Met genotypes and metabolic risk parameters stratified by lipid status, N, normolipidemia; H, hyperlipidemia. Other notes and abbreviations see legends of Fig. 3. (JPG 1289 kb) [file 12877_2016_393_MOESM3_ESM.jpg]
